# Pulsed electric fields: a sharp sword in the battle against cancers

**DOI:** 10.3389/fimmu.2025.1682539

**Published:** 2025-11-20

**Authors:** Lu Zhang, Shoulong Dong, Fei Teng, Yuan Wang, Wei Xu, Yang Chen, Liang Yu, Chenguo Yao, Zhiqiang Wang

**Affiliations:** 1 Center of Thoracic Cancer, Chongqing University Cancer Hospital, Chongqing, China; 2 Chongqing Key Laboratory for the Mechanism and Intervention of Cancer Metastasis, Chongqing, China; 3 State Key Laboratory of Power Transmission Equipment Technology, School of Electrical Engineering, Chongqing University, Chongqing, China

**Keywords:** pulsed electric fields, cancer, irreversible electroporation, immunotherapy, mechanism of action, clinical prospects

## Abstract

Cancer remains a significant threat to human health, and conventional treatments such as surgery, chemotherapy, and radiotherapy have their limitations. In recent years, pulsed electric fields (PEFs) has garnered attention as an emerging method for cancer treatment. It primarily utilizes high-intensity pulse electric fields applied to tumor cells, inducing effects such as electroporation or internal electrical processing, which lead to cell death. This review will introduce the principles of PEFs, its application fields, and its prospects in cancer treatment, aiming to provide readers with a comprehensive understanding of the research areas related to PEFs and cancer therapy.

## 1 Introduction

Cancer treatment remains a complex and highly challenging field despite significant progress over the past few decades. Key obstacles include tumor heterogeneity, where diverse cell subpopulations and genetic variations lead to different treatment responses, complicating strategy development (1). Drug resistance arises as tumor cells evade therapy through mechanisms like gene mutations, altered drug transport, and activated survival pathways, reducing treatment efficacy (2). Toxic side effects from traditional therapies like chemotherapy and radiotherapy damage healthy cells, causing severe burdens such as nausea, vomiting, hair loss, and fatigue (3). The intricate tumor microenvironment (TME), comprising tumor cells, blood vessels, immune cells, and extracellular matrix, forms an ecosystem that significantly impacts treatment effectiveness and complicates design (4). Early diagnosis and treatment, crucial for success, are hindered by the frequent absence of symptoms in early-stage tumors and the highly invasive behavior of some cancers (5). Finally, individual differences in tumor biology and patient characteristics render standardized approaches inadequate, necessitating personalized strategies focused on genomics and molecular profiles for customized care (6).

In the face of these challenges, scientists and physicians are actively exploring new treatment strategies and technologies. First of all, physical therapy offers significant advantages. Pulsed electric fields (PEFs), as an emerging method in physical therapy for cancer treatment, offers unique advantages and promising applications, potentially providing new solutions to overcome the challenges in cancer therapy (7–9). PEFs, as an emerging method for cancer treatment, originated from the electroporation technology of the 1980s. Initially, electroporation was used as a research tool to enhance cellular membrane permeability. By applying high-intensity PEFs, transient pores are created in the cell membrane, allowing large molecules such as drugs or DNA to enter the cell (10). As research into electroporation deepened, it was realized that the effect of PEFs on cells was not only to increase membrane permeability but could also directly destroy the integrity of the cell membrane, leading to cell death-a phenomenon known as irreversible electroporation (IRE) (11). IRE have become hot topics in the field of cancer treatment among scholars (11–15). The earliest experiments with PEFs therapy were conducted in mouse tumor models and showed that PEFs could significantly inhibit tumor growth (16). Subsequent studies indicated that PEFs could induce tumor cell death through various mechanisms, including membrane electrical breakdown (17), apoptosis (18), cell cycle arrest (19), and activation of immune responses (20). With further technological development, researchers began to explore the application of PEFs in clinical cancer treatment. The first clinical trials applying PEFs to human cancer treatment were conducted in the 1990s, primarily targeting localized lesions such as skin tumors (21, 22). These trials demonstrated that PEFs could effectively control and reduce tumor volume. As the technology has continued to improve and clinical research has deepened, the application scope of PEFs has gradually expanded to include various types of solid tumors and hematological malignancies (23, 24). Additionally, PEFs can be combined with other treatments such as chemotherapy (25), radiotherapy (26), and immunotherapy (27) to enhance therapeutic efficacy.

**Figure 1:**
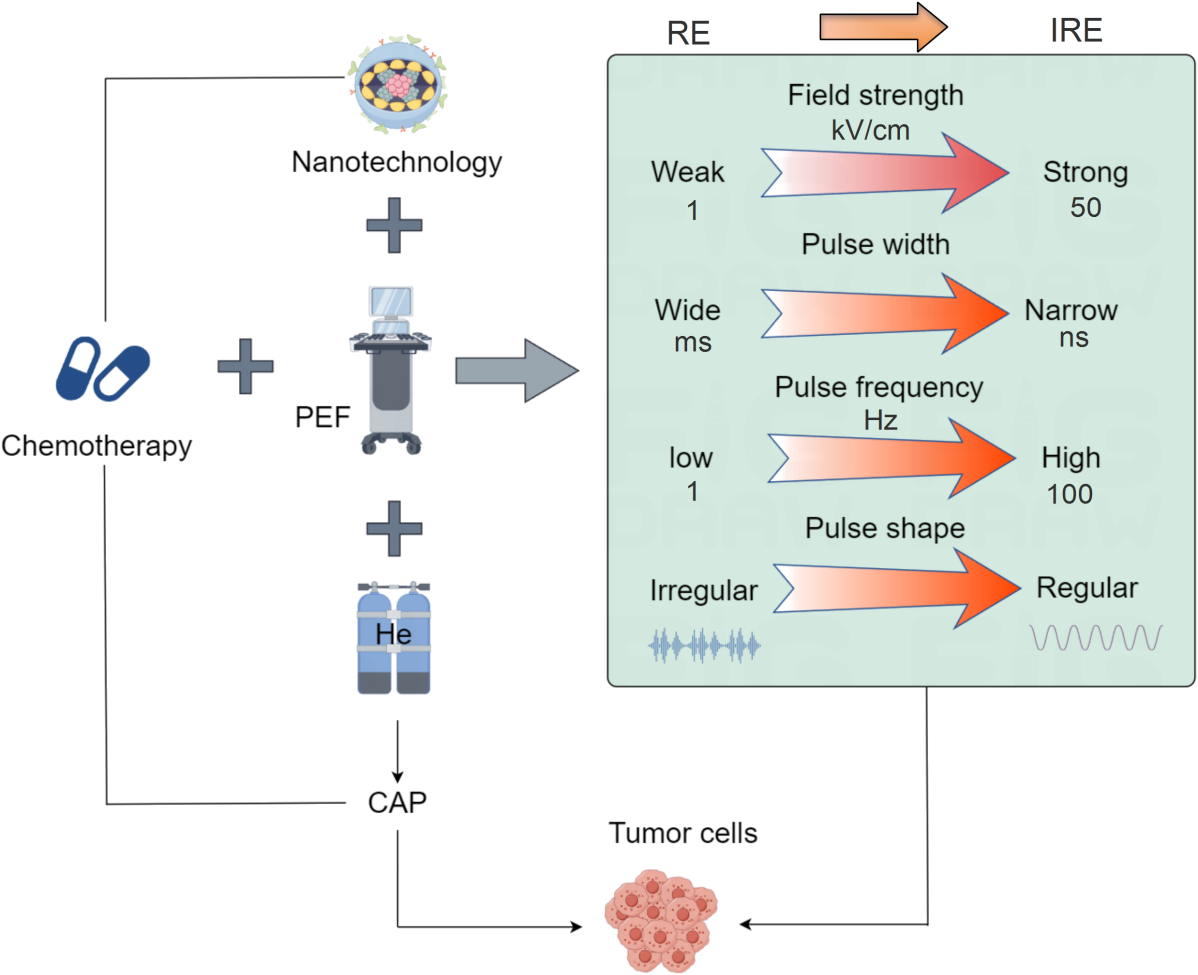
Schematic diagram of PEFs technology development. The application of PEFs technology in oncology has evolved from reversible electroporation to irreversible electroporation. The key parameters involved in PEFs have also progressed: the electric field intensity has increased from low (1 kV/cm) to high (over 50 kV/cm), the pulse width has shortened from long (ms) to short (ns), the frequency has risen from low (1 Hz) to high (over 100 Hz), and the pulse shape has become more regular from irregular. The development of PEFs combined with other modalities includes integration with radiotherapy and chemotherapy, combination with nanomaterials, pairing with cold atmospheric plasma (CAP), and coupling with immunotherapy, ultimately achieving targeted minimally/non-invasive tumor treatment.

In summary, the development background of PEFs can be traced back to the research on electroporation, which has gradually evolved into a unique method for cancer treatment. With further research and clinical practice, PEFs is expected to become one of the important means of cancer treatment, offering patients more effective and personalized treatment options (**Figure 1**).

## 2 Working principles and treatment modalities of PEFs in tumor therapy

### 2.1 The working principles of PEFs

PEFs refers to a high-voltage pulse applied over an extremely short period of time. It is a special type of electrical signal characterized by high electric field strength and a brief duration of action. In cancer treatment, PEFs are used to directly target tumor tissue to achieve therapeutic objectives.

The core principles governing PEFs encompass several critical parameters. Electric field strength–measured in kilovolts per centimeter (kV/cm)–serves as a pivotal determinant, where elevated intensities induce electrical breakdown and irreversible membrane disruption to eliminate tumor cells. Pulse width, quantified in nanoseconds (ns) or microseconds (μs), directly influences thermal and electrolytic side effects; shorter durations enhance selectivity for neoplastic cells while minimizing collateral damage. Shorter pulse widths and wavelengths deliver higher energy, which penetrates the outer cell membrane to directly target internal structures. Consequently, adjusting these parameters allows for selective targeting of internal cellular components. Pulse frequency, expressed in hertz (Hz), must be optimized according to therapeutic objectives and tumor-specific characteristics. Additionally, pulse waveform variations–including square, exponential decay, and triangular configurations–modulate cellular interactions and biological outcomes, enabling tailored bioelectrical effects. Collectively, these parameters define PEFs’s mechanism of action through controlled electroporation.

In cancer treatment, PEFs acts on tumor tissue by applying high-intensity pulsed electric fields, producing electrical breakdown and destructive effects on the cell membrane, known as irreversible electroporation (IRE). As opposed to reversible electroporation (RE), which is characterized by lower field strength, wider pulses, and lower frequency. Its action is confined to the outer cell membrane, enabling membrane recovery and thus preventing cell death (**Figure 1**). This disrupts the ionic balance inside and outside the cell, increases membrane permeability, impairs the exchange of substances across the membrane, and ultimately leads to the death of tumor cells. Additionally, PEFs can activate the immune system to promote an immune response against the tumor (28, 29). It is important to note that the specific application methods and parameter choices of PEFs in cancer treatment may vary depending on the type of tumor, treatment goals, and individual patient differences (9). Therefore, doctors will determine the appropriate PEFs parameters and treatment plans based on the patient’s condition and treatment needs for specific cases.

### 2.2 The strategies of PEFs in tumor treatment

PEFs employs electric field energy to target tumors through diverse implementation approaches tailored to specific therapeutic objectives (**Table 1**). In its standard form, PEFs therapy applies intra- or extracorporeal electric fields using micro-to-submicrosecond pulses at kV/cm strengths, inducing cell membrane electroporation to trigger apoptosis, necrosis, or cell cycle arrest for tumor suppression. Electrochemotherapy (ECT) synergistically combines PEFs with chemotherapeutic agents: drugs are administered intratumorally before PEFs application, where transient membrane permeabilization enhances intracellular drug uptake, significantly potentiating cytotoxicity against treatment-resistant malignancies. Electrohyperthermia (EHT) utilizes high-frequency currents to generate localized thermal effects, either directly ablating tumor cells to augment radiotherapy/chemotherapy efficacy. Alternatively, Electroporation-Mediated Gene Therapy (EMGT) leverages PEFs to reversibly permeabilize membranes, enabling targeted delivery of genetic payloads (therapeutic genes/DNA constructs) for precision genome editing, cellular reprogramming, or molecular interventions (**Figure 1**).

**Table 1:** The application of PEFs in different solid tumors.

Tumor types	Form of PEFs	Characteristics	Target	Effect	Mechanism of action	References
Pancreatic cancer	♦ IRE ♦ nsPEF	Acts on all cellular membrane structures without selectivity; Acts selectively on the intracellular membranes.	Animal: Orthotopic pancreatic cancer models, Panc02 tumors	**+++**	♦ Immune regulation ♦ mRNA-miRNA-lncRNA network ♦ DDR	(18–34, 87, 88)
HCC	♦ IRE ♦ nsPEF	Acts on all cellular membrane structures without selectivity; Acts selectively on the intracellular membranes.	Animal: Rabbit VX2 liver tumor, Tumor-bearing mouse Cell: Hepa1-6 and HepG2	**+++**	♦ Immune regulation ♦ Gut microbiome	(15, 35–43, 45–48)
Melanoma	♦ nsPEF, ♦ nsPMFs	Acts selectively on the intracellular membranes; Without selectivity.	Patient: Vulvar melanoma patient Animal: B16F10 melanoma mice, Horses presenting with spontaneous melanoma Cell: A375 and C32 melanoma cells	**++**	♦ Apoptosis ♦ Immune regulation	(56, 57, 59–63)
Prostate cancer	♦ IRE, ♦ nsPEF, ♦ H-FIRE ♦ PEMF	Without selectivity; Acts selectively on the intracellular membranes; Without selectivity; Without selectivity.	Animal: Mouse model of prostate carcinoma, Cell: PCa cells, PC3	**+++**	♦ Immune regulation ♦ Apoptosis	(49, 50, 52–54, 89, 90)
Breast cancer	♦ nsPEF ♦ PEF ♦ H-FIRE	Acts selectively on the intracellular membranes; Without selectivity; Without selectivity.	Animal: 4T1 mammary mouse model Cell: MCF-7, 4T1	**++**	♦ CXCL9 axis ♦ Immune regulation ♦ Apoptosis	(70–76)
Glioblastoma	♦ H-FIRE ♦ nsPEF	Without selectivity; Acts selectively on the intracellular membranes.	Animal: Orthotopic tumor-bearing glioma model Spheroids: C6, and GL261 spheroids Cell: CSC, F98 glioma, U87-MG cells	**++**	♦ Apoptosis ♦ Immune regulation	(65–69)
Lung cancer	♦ H-FIRE ♦ CaEP	Without selectivity; Without selectivity.	Patient: Patients with early-stage NSCLC Cell: A549	**+**	♦ Oxidative stress ♦ Apoptosis	(13, 77–80)
Colorectal cancer	♦ nsPEF	Acts selectively on the intracellular membranes.	Cell: CT-26, EL-4, LS 174T, HT-29	**+**	♦ Endoplasmic reticulum stress ♦ ICD ♦ Apoptosis	(82, 83)
Ovarian cancer	♦ nsPEF ♦ BEP	Acts selectively on the intracellular membranes; Without selectivity.	Cell: Mouse ovarian surface epithelial, OvBH-1	**+**	♦ Apoptosis	(85, 86)
Head and neck cancer	♦ IRE	Without selectivity.	Animal: Head and neck cancer xenografts	**+**	♦ PI3K/mTOR	(84)
Gastric cancer	♦ nsPEF	Acts selectively on the intracellular membranes.	Cell: Gastric adenocarcinoma cell lines	**+**	♦ Oxidative stress ♦ Apoptosis	(81)

DDR, DNA damage repair responses; PEMF, Pulsed electromagnetic field; H-FIRE, High frequency irreversible electroporation; CaEP, Calcium electroporation; IRE, Irreversible electroporation; nsPEF, Nanosecond pulsed electric field; nsPMFs, Nanosecond pulsed magnetic fields; OvBH-, Ovarian clear carcinoma cell line; CSC, Cancer stem cells; ICD, Immunogenic cell death; BEP, Bipolar electroporation; HCC, Hepatocellular carcinoma. “+” indicated significant effect, and more plus signs correlate with improved treatment outcomes for this type of tumor.

Collectively, these modalities demonstrate PEFs’ adaptability across mechanistic paradigms—from physical ablation to molecular-scale interventions.

## 3 The application of PEFs technology in cancer treatment

### 3.1 Pancreatic cancer

Pancreatic cancer stands as one of the malignancies most frequently targeted by PEFs therapy, which not only significantly suppresses tumor progression but also remodels the immunosuppressive TME, thereby preventing recurrence and metastasis. For example, IRE delivers high-voltage electric pulses to disrupt cellular membrane integrity, inducing immunogenic cell death (ICD) through damage-associated molecular pattern (DAMP) release and loss of intracellular homeostasis (30). In preclinical models, IRE monotherapy suppresses pancreatic tumor growth in a dose-dependent manner while enhancing CD8^+^ T-cell infiltration (30, 31). Its immunomodulatory capacity—evidenced by HMGB1-mediated MAPK-p38 activation and M1-macrophage polarization (32)—synergizes with immunotherapies: combined IRE and CD40 antibody therapy stimulates systemic immunity and inhibits metastasis in orthotopic models (31); IRE/OX40 agonist regimens enhance CD8^+^ T-cell quality/quantity and extend survival (33); and IRE-induced interferon-γ expression supports checkpoint inhibitor combinations to prevent recurrence (34). IRE further overcomes immunosuppression in the TME, improving dendritic cell vaccine efficacy (35) and promoting CD4^+^ T-cell conversion to antitumor Th1/Th17 subsets in MHC-I-low PDAC, where subsequent PD-L1 blockade activates compensatory cDC2-CD4^+^ T-cell axes (36). Advanced delivery strategies amplify these effects—nanoparticles loaded with TGF-β inhibitors (SB525334) or gemcitabine (NE/Lip-GEM) enhance neutrophil-mediated drug delivery (27, 37, 38), while M-TK-OA nanotherapeutics combined with IRE activate cGAS-STING pathways to induce durable immune memory via PARP/ATM inhibition (39). Clinically, adding PD-1/PD-L1 blockers to IRE-chemotherapy regimens improves survival in locally advanced pancreatic cancer (LAPC) (40). Complementary electrical modalities like nsPEF inhibit growth/metastasis while remodeling myeloid compartments (41–43), and electro-antibacterial therapy (EAT) enhances intracellular pathogen clearance (44). Collectively, IRE-based combinatorial approaches reshape the TME toward proinflammatory states (19), establishing a promising paradigm for immunologically resistant pancreatic malignancies.

### 3.2 Hepatocellular carcinoma

Clinical studies have established IRE as a safe and effective hepatocellular carcinoma (HCC) ablation modality, with ongoing optimization of efficacy assessment methods and device engineering complementing robust evidence of its antitumor effects. Preclinical validation includes rabbit HCC models demonstrating significant histological advantages of IRE over RE and untreated controls: IRE zones exhibited 51-60% fewer tumor cells, 66-67% reduced microvasculature, and 185-228% increased cell death (45). Technical refinements further define therapeutic parameters—thresholds for near-complete HepG2 cell eradication require IRE pulses at 4kV/cm, while RE permeabilization occurs at 1kV/cm (8 pulses) with maximum temperatures ≤30.1 °C, confirming non-thermal mechanisms (46). nsPEF demonstrates compelling preclinical efficacy: it inhibits Hep3B cell growth *in vitro* with distinct ultrastructural changes observed via TEM, achieves complete ablation in rabbit VX2 liver tumors under contrast-enhanced ultrasound guidance (47), and alters osteopontin-mediated glycogen metabolism in HCC (48). Crucially, nsPEF induces immunogenic cell death that stimulates systemic immunity against residual/metastatic disease (49), though it simultaneously elevates membrane PD-L1 levels and promotes PD-L1^+^ extracellular vesicle release, causing CD8^+^ T-cell dysfunction—an effect reversed by PD-L1 blockade to significantly suppress tumor growth and improve survival (50, 51). IRE monotherapy in H22 murine models similarly enhances CD8^+^ T-cell and dendritic cell infiltration in peri-ablational zones (52), while IRE-immunotherapy combinations in orthotopic models remodel tumor immunity by enhancing infiltrating CD8^+^ T cell necroptosis yet attenuating pro-tumor inflammatory populations (15). Clinical translation progresses rapidly: nsPEF safely alters sphingolipid metabolism to drive Ly6c2^+^ mononuclear phagocyte differentiation and memory CD8^+^ T-cell formation in human trials (53), while ultrasound-guided IRE fulfills clinical guidelines by enabling tumor control, symptom alleviation, and survival extension (54). Emerging combinatorial approaches include IRE with Chlorella vulgaris/polydopamine-encapsulated PD-1 inhibitors to boost local drug concentration and immune activation (55), and high-frequency repetitive nsPEF (rnsPEF) which achieves effective ablation at 4536.4 ± 618.2 V/cm in rabbit livers with preserved vasculature. rnsPEF synergizes with doxorubicin to enhance cell death and long-term tumor control (56), reinforcing nsPEF’s preclinical safety profile for high-risk HCC locations (57). Microbiome and serum metabolome analyses further suggest novel prognostic markers post-ablation (58). Collectively, these advances establish PEFs—particularly through IRE and nsPEF platforms—as transformative tools that overcome limitations of thermal ablation, with clinical integration accelerated by standardized protocols, immune modulation strategies, and precision energy delivery systems.

### 3.3 Prostate cancer

Emerging clinical evidence establishes IRE as a safe and effective modality for prostate cancer treatment, with recent studies highlighting its capacity to potentiate checkpoint immunotherapy. Specifically, IRE enhances systemic antitumor T-cell activation while downregulating immunosuppressive mechanisms in localized disease, promoting tumor antigen-specific expansion of tissue-resident memory CD8^+^ T cells (TRM) for durable immune surveillance (20, 59). Complementary nsPEF technology exerts distinct cytotoxic effects: high-frequency nsPEF induces profound cytoskeletal alterations that disrupt cellular mobility and enhance membrane permeability, ultimately triggering prostate cancer cell death (60). Parametric optimization studies in murine models demonstrate voltage-dependent efficacy, with histological, immunohistochemical, and immunoblot analyses confirming that 900 V represents the minimal threshold voltage for significant tumor growth reduction via IRE-induced cell death (61). *In vitro* and magnetic resonance imaging (MRI) assessments further validate IRE’s ability to achieve sustained tumor regression (62). When combined with microwave ablation, PEFs synergistically reduce PC3 cell viability, induce apoptosis, and inhibit migratory capacity—as quantitatively demonstrated through scratch assays (63). Current reviews comprehensively summarize these advances, analyzing IRE’s mechanisms, clinical outcomes, advantages, and limitations while highlighting critical research gaps and future directions for optimizing PEFs-based prostate cancer management (14). Collectively, these findings position IRE and nsPEF as transformative tools that bridge focal ablation with systemic immunomodulation in prostate oncology.

### 3.4 Melanoma

PEFs represent a highly suitable therapeutic approach for melanoma, primarily because these tumors are typically located in superficial layers, allowing for direct percutaneous minimally invasive intervention that yields clinically significant outcomes. For instance, nsPEF exert tumor-ablation effects primarily through subcellular membrane electroporation, offering cell-type specificity with minimal thermal damage while synergizing with chemotherapeutics—positioning it as a promising modality for melanoma treatment (64). Preclinically, nsPEF-mediated local ablation in murine melanoma models restores (though does not enhance) dormant antitumor immunity in tumor-bearing hosts (65), while nsPEF treatment induces non-cytotoxic membrane permeabilization and morphological changes characterized by vesicle externalization, cell contraction, and lipid migration. Critically, this elevates PD-1 checkpoint expression, suggesting therapeutic synergy with immune checkpoint inhibitors (66). Nanosecond pulse stimulation (NPS) demonstrates superior efficacy over cryoablation, permanently eliminating up to 91% of B16-F10 melanoma lesions (vs. 66% with cryoablation) with minimal fibrosis, muscle atrophy, or permanent skin damage—establishing it as a less destructive yet more effective alternative (67). Complementary approaches include high-frequency nsPEF combined with magneto-poration of iron oxide nanoparticles to suppress A375 cell viability *in vitro* (68), and integrin-targeted nsPEF IRE (INSPIRE) achieving voltage-dependent tumor reduction in equine spontaneous melanoma (84-88% volume reduction at 2kV) (69). Clinically, PEFs-mediated mRNA delivery of cyclin B1 knockdown induces tumor regression (70), while nsPEF upregulates melanoma-specific MAGE antigen expression to sensitize tumors to targeted therapies (71). Early human trials report rapid resolution of immunotherapy-resistant uveal melanoma metastases following PEFs treatment (72), underscoring its translational potential. Collectively, these modalities leverage unique bioelectrical mechanisms—from subcellular electroporation to immune checkpoint modulation—to overcome therapeutic resistance in melanoma.

### 3.5 Gliomas

For gliomas, PEFs (specifically IRE and nsPEF) effectively address the limitation of traditional chemotherapeutic agents being unable to penetrate the blood-brain barrier (BBB), enabling targeted treatment of tumor lesions while minimizing collateral damage to healthy brain cells (73). Studies utilizing high-intensity ultrashort PEFs on U87 GBM cells and U87-derived neurospheres revealed, through the analysis of diverse *in vitro* biological endpoints and transcriptomic and bioinformatic analyses, that PEFs affects cell proliferation, differentially regulates hypoxia, inflammation, and p53/cell cycle checkpoints, significantly reduces the capacity to form new neurospheres, inhibits invasive potential, and alters the expression of stemness/differentiation genes (74). High-frequency irreversible electroporation (H-FIRE) was applied to suspensions of F98 glioma and LL/2 Lewis lung carcinoma cells to model primary and metastatic brain cancer. Data indicate that H-FIRE induces both reversible and irreversible cell damage in a dose-dependent manner, and the existence of dose-dependent recovery mechanisms allows tumor cell proliferation (75). Furthermore, H-FIRE has been shown to improve survival and immune cell infiltration in rodent models of malignant glioma (76). Through cell ablation and survival experiments, this study investigated the bipolar cancellation (BPC) effect in U87-MG cells exposed to nsPEF with varying pulse numbers and electric field amplitudes. Results demonstrated the highest BPC efficiency (163.9%) occurred at 15 kV/cm and 15 pulses, while unipolar nsPEF at 20 kV/cm and 15 pulses achieved 90% lethality in cell suspensions; this latter field was subsequently used as the reference for ablation experiments. Ablation studies revealed that the electric field threshold for ablation was lower in 3D (3D-like tissue) models (5.805 ± 1.455 kV/cm) compared to monolayer walled cells (8.95 ± 0.75 kV/cm), resulting in larger ablation areas under identical pulse conditions. Additionally, the BPC effect was more pronounced for 3D cells, although ablation area and BPC efficiency followed similar trends when pulse number was modulated (77). Collectively, this work highlights the potential of ultrashort pulsed electric fields to disrupt the dense structure of glioma (78).

### 3.6 Breast cancer

PEFs effectively overcome chemoresistance in breast cancer while offering a minimally invasive, safe, and efficacious therapeutic approach. For example, this study aimed to evaluate the treatment of MCB-7 human breast cancer cells using nsPEF and low electric fields (LEFs) unipolar electrical pulses. At repetitive frequencies starting from 0.01 Hz, cell viability was significantly reduced by approximately 35%, reaching complete cell loss with microsecond pulses at 1 Hz. Uptake of non-permeant drugs occurred not via classical electroporation-mediated membrane permeabilization, but through endocytosis. Microsecond electric pulses were able to disrupt the membranes of endocytotic vesicles, releasing the cytotoxic drug bleomycin (79). PEFs treatment combined with azithromycin significantly inhibited human breast cancer cell proliferation (80). nsPEF effectively ablated tumors, elicited an immune response, and suppressed residual breast cancer growth in mice via a CXCL9 axis-dependent mechanism (81). Treating orthotopic breast cancer-bearing mice with PEF revealed significant immunomodulatory effects compared to radiofrequency ablation (RFA). Distinct serum and tumor cytokine profiles were observed, including intratumoral downregulation of vascular endothelial growth factor (VEGF), hypoxia-inducible factor 1 alpha (HIF-1α), c-MET, interleukin-10 (IL-10), Ki67, and tumor necrosis factor alpha (TNF-α). PEFs increased innate immune activation, enhancing the recruitment of dendritic cells, M1 macrophages, and natural killer cells, while decreasing M2 macrophages and myeloid-derived suppressor cells. Concurrently, PEFs enhanced adaptive immunity compared to RFA, characterized by increased antigen-specific T cells and reduced regulatory T cells. By the study endpoint, PEFs suppressed tumor growth and increased survival. Finally, PEFs promoted an abscopal effect clearing lung metastases, with the effect being stronger when combined with anti-PD-1 therapy than PEFs alone (29). Research applying nsPEF to electrically stimulate breast cancer MCF-7 cells demonstrated that nsPEF distinctly impacted intracellular functions and dynamics in both MCF-7 and MCF-10A cells. This study proved the selective killing of breast cancer cells using microelectrodes, paving the way for developing nsPEF-based breast cancer therapies (82). This work investigated how sub-ablative H-FIRE affects lymphatic and blood microvascular remodeling in the 4T1 murine breast cancer model. Histological examination revealed a transient increase in blood vessel density on day 1 post-treatment, followed by a peak in lymphatic vessel density within surviving tumor regions on day 3, alongside increased lymphatic vessel density in the surrounding fat pad; minimal remodeling occurred in tumor-draining lymph nodes within 3 days. Gene expression analysis indicated elevated CCL21 and CXCL2 levels on day 1, while VEGFA and VEGFC did not appear to drive vascular remodeling. Similarly, CCL21 protein content in tumor-draining axillary lymph nodes correlated with gene expression data from surviving tumor regions. These findings demonstrate dynamic changes in lymphatic and blood microvascular architecture post-SA-HFIRE, potentially enhancing adaptive immune responses through CCL21-mediated lymphatic homing and subsequent lymph node microvascular remodeling (83). Finally, we demonstrate that μsPEF induces MCB-7 cell death through a mechanism dependent on Ca^2+^ electropermeation (CaEP) and calpain activity (84).

### 3.7 Other cancers

Beyond the malignancies mentioned above, PEFs demonstrate robust therapeutic efficacy in lung carcinoma, colon carcinoma, head and neck carcinoma, gastric carcinoma, and ovarian carcinoma, among others. Firstly, high-frequency irreversible electroporation (H-FIRE) treatment of primary lung tumors in dogs revealed tumor ablation shrinkage, and immunohistochemical staining results suggested H-FIRE may alter the tumor immune microenvironment (13). Combining high-frequency unipolar nsPEF with Ca^2+^ inhibited lung cancer cells growth (85). Clinical trials indicate that PEFs therapy is feasible and safe for early-stage non-small cell lung cancer (NSCLC), showing potential signals of immune system activation (86). Furthermore, PEFs treatment improved progression-free survival and overall survival (OS) in patients with progressive stage IV NSCLC (87). Calcium ions and optimized pulse parameters can enhance PEFs efficacy and induce oxidative changes in lung cancer cells. Therefore, the anticancer efficacy of PEFs combined with standard cytotoxic drugs or calcium ions should be considered for lung cancer treatment (88). Secondly, combining PEFs with daunorubicin in two gastric adenocarcinoma cell lines (ERG85-257P and ERG85–257 RDB) inhibited proteasome activity, leading to increased protein degradation, elevated cell death percentage, and heightened reactive oxygen species (89). Thirdly, nsPEF induced endoplasmic reticulum stress accompanied by immunogenic cell death in murine lymphoma and colorectal cancer models (90). nsPEF treatment reduced viability, proliferation, and mucin production in mucinous colorectal cancer (MCRC) cells while promoting cell death, indicating its potential clinical application for MCRC (91). Fourthly, the combination of IRE and L-BEZ effectively eradicated tumors and prevented recurrence in nude mice bearing head and neck tumors (24). Electrochemotherapy involves treating solid tumors by combining non-permeant cytotoxic drugs (e.g., bleomycin) with locally applied PEFs. Effective use of this method crucially depends on utilizing optimal PEFs protocols and concentrations of drugs that lack inherent cytotoxicity (25). Fifthly, results demonstrate that nsPEF can exert preferential ablation effects on highly invasive and malignant ovarian cancer cells compared to benign cells. This study provides an experimental foundation for research into killing malignant cells via electrotherapy and may hold clinical significance for tumor treatment and preventing post-treatment recurrence (92). Research shows that pulse asymmetry influences the bipolar cancellation (BPC) effect. Following Ca^2+^ electrochemotherapy, cell membrane poration decreased while cell death increased. The BPC phenomenon can be controlled using pulse asymmetry or a delay between the positive and negative phases of the pulse (93).

## 4 The mechanisms of PEFs in promoting cancer cells death

PEFs, including nsPEF, IRE, and H-FIRE, have been demonstrated to effectively induce cancer cell death through multiple mechanisms. Among these, IRE primarily promotes tumor cell death through its characteristic effects. However, due to its lower energy and shorter pulse width, RE often results in transient, repairable membrane permeabilization that is insufficient to cause tumor cell death. The cancer cells that survive IRE treatment, or those targeted by nsPEF, can subsequently undergo death via pathways such as apoptosis (94–98), autophagy (99, 100), and ferroptosis (101, 102).

Fundamental studies indicate that high-intensity electric pulses can significantly increase cell membrane permeability. Molecular dynamics simulations and free energy calculations reveal that this membrane permeabilization lesion undergoes secondary oxidation, inducing substantial lipid peroxidation that triggers ferroptosis (101). In various cell models, PEFs induce programmed cell death. For example, nsPEF treatment of human ovarian cancer cells (SKOv3) may induce apoptosis by activating the release of intracellular calcium stores (95); nsPEF significantly reduces viability, inhibits growth, and induces apoptosis in prostate cancer cells (PPC-1) (94); and IRE treatment of rat tissues activates caspase-3 and promotes apoptosis, as observed via immunohistochemistry and TUNEL assay (97). Furthermore, research on glioma cells (U251) shows that PEFs not only mediate apoptosis via the AP-1/Bim pathway but also involve other forms of regulated cell death (RCD), such as autophagy, necroptosis, and immunogenic cell death (ICD) (96).

Autophagy plays a complex role in the effects of PEFs. For instance: Sub-toxic doses of nsPEF activate autophagy as a compensatory mechanism to repair membrane damage, but prolonged exposure increases cell death while concomitantly reducing autophagy markers (99). Conversely, in a pancreatic cancer model using KPC-A548 or Panc02 murine cell line xenografts, IRE increased the expression of autophagy markers LC3 and p62. Inhibiting autophagy (e.g., with hydroxychloroquine) significantly enhanced the efficacy of IRE. Subsequent combination of IRE with inhibitors targeting both HMGB1 receptors (RAGE and TLR4) further suppressed tumor growth, indicating that IRE promotes cell death, in part, by modulating autophagy (100).

Notably, nsPEF can also exert cell cycle-specific effects on tumor cells, such as inhibiting the proliferation of S-phase cells without significantly affecting their viability (103). Importantly, PEFs can produce synergistic effects with traditional chemotherapy. For example, the chemotherapeutic agent 5-fluorouracil (5-FU) demonstrated effectiveness when combined with nsECT (nanosecond electrochemotherapy) and Fe(III). This combined approach significantly reduced the expression of the mitochondrial protein frataxin under a microsecond electroporation protocol, inducing ferroptosis (102). Additionally, H-FIRE was shown to inhibit the invasion and metastasis of highly aggressive tumor cells by suppressing SIRT1/2 expression and inducing mitochondrial cell death (98).

**Figure 2:**
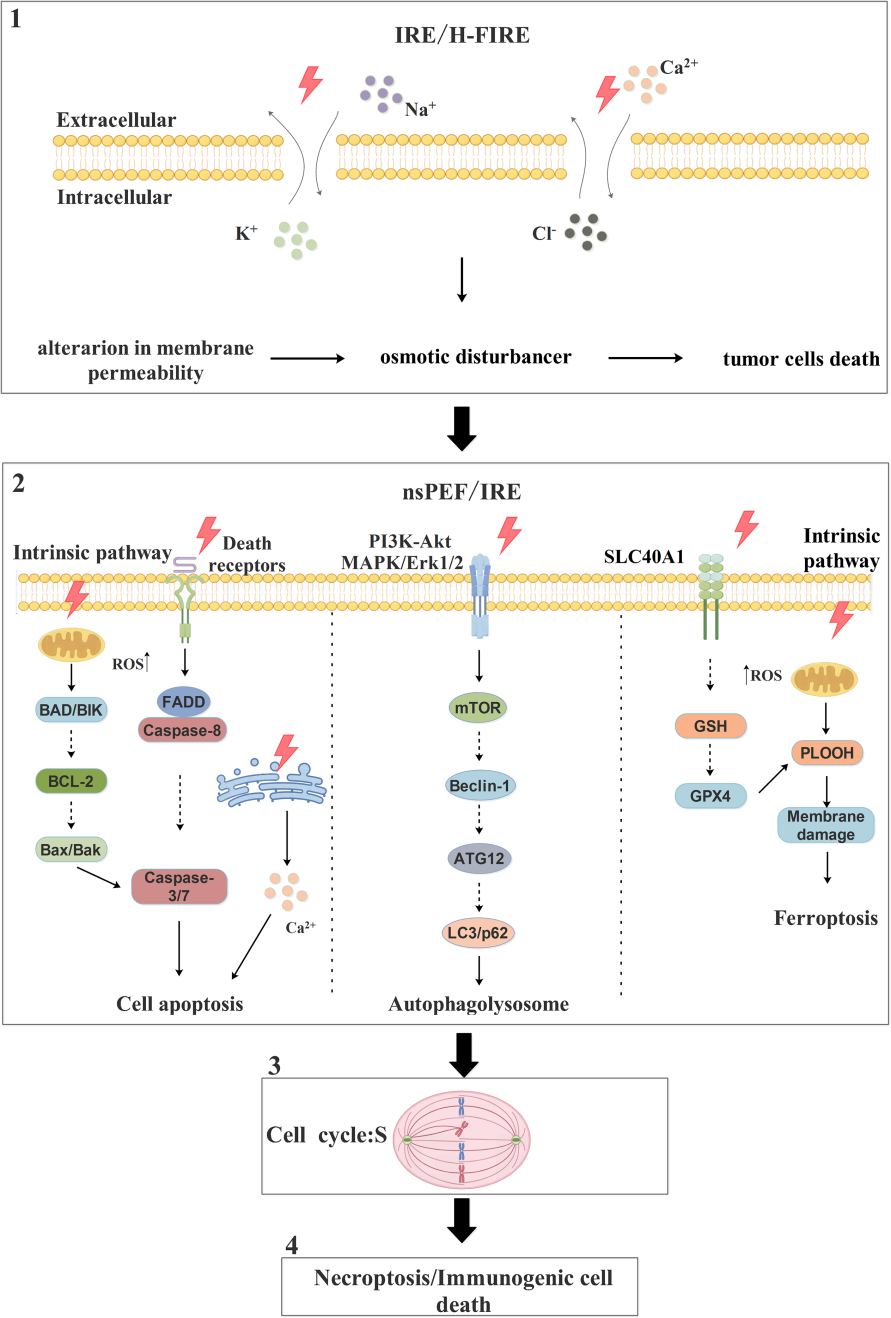
The pathways and molecular mechanisms of PEFs in promoting tumor cells death. PEFs primarily promote tumor cells death through three main pathways: apoptosis, autophagy, and ferroptosis. In apoptosis, one mechanism involves PEFs inducing an increase in endogenous ROS levels, which activates downstream pro-apoptotic proteins BAD and BIK; this activation inhibits the anti-apoptotic protein BCL-2, leading to the release of Bax and Bak, which subsequently activate caspase-3/7 and trigger apoptosis. Alternatively, PEFs promote apoptosis by triggering death receptors on the cell surface, leading to the recruitment of the adaptor protein FADD and the activation of caspase-8; this in turn activates caspase-3/7, resulting in substrate protein hydrolysis and cell death. Autophagy is induced by PEFs influencing the PI3K-Akt and MAPK/Erk1/2 signaling pathways to activate the downstream mTOR pathway; this activation promotes the formation of the Beclin-1/ATG12/LC3 complex, leading to autophagosome generation, fusion with lysosomes, and ultimately cell death. For ferroptosis, one mechanism involves PEFs affecting the metal transporter SLC40A1 to increase Fe²^+^ uptake; this increased iron influx inhibits the GSH/GPX4 antioxidant system, resulting in the accumulation of large amounts of phospholipid hydroperoxides (PLOOH), which disrupt membrane integrity and induce ferroptosis. Note: Solid lines indicate reported cases, while dashed lines indicate unreported cases.

Collectively, these studies reveal the potential of PEFs to exert multi-faceted anti-tumor effects by inducing apoptosis, modulating autophagy, triggering ferroptosis, activating other RCD pathways, and potentially synergizing with chemotherapeutic drugs (**Figure 2**). Furthermore, different PEFs modalities trigger distinct cell death pathways. For instance, nsPEF primarily induces cell death through apoptosis, autophagy, and ferroptosis. In contrast, IRE can not only initiate these same pathways but also disrupt the ionic balance across both the inner and outer cell membranes to cause cell death.

## 5 Immune regulatory mechanisms of PEFs in the TME

**Figure 3:**
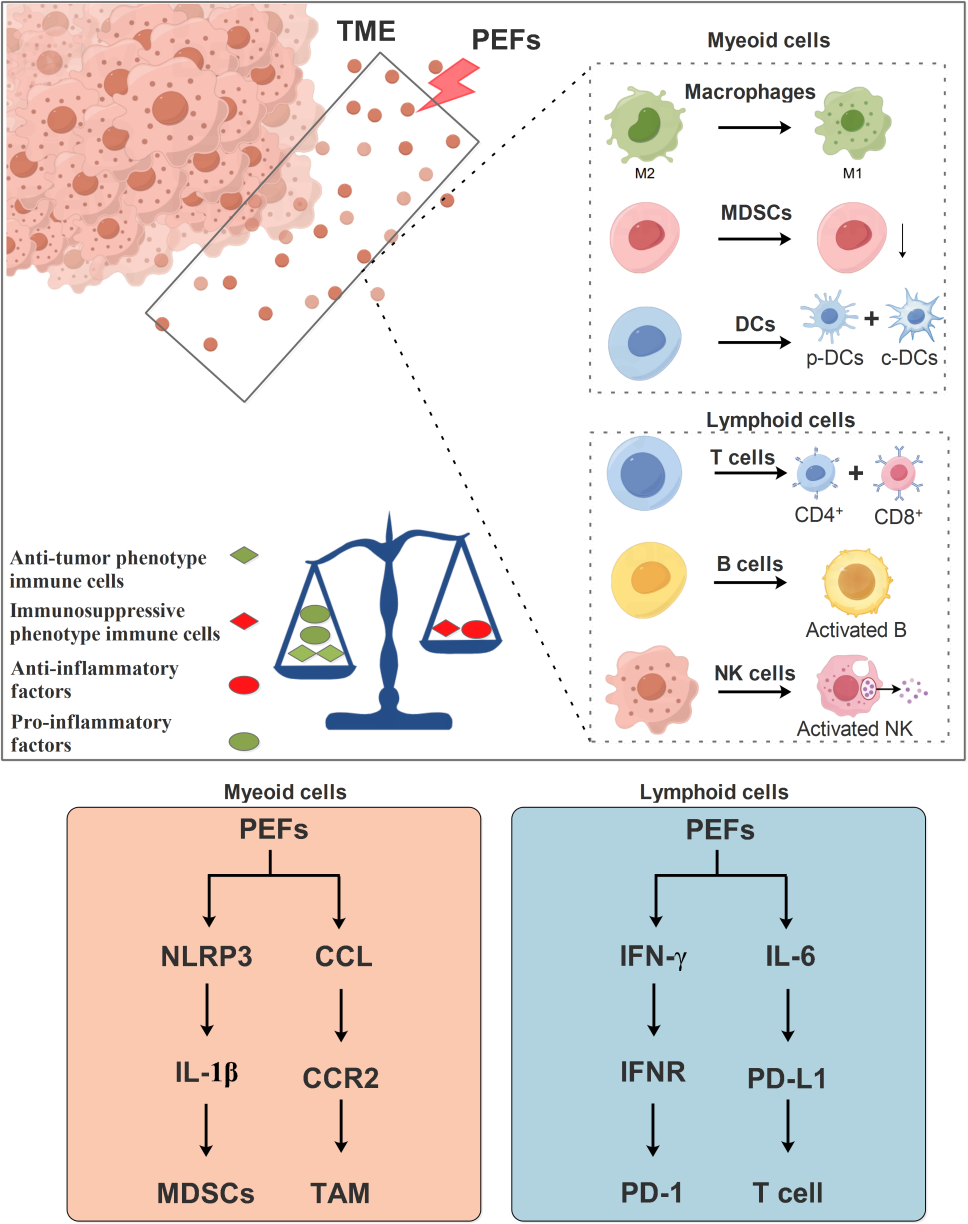
Mechanism of PEFs in regulating immune responses within the TME. PEFs influence immune cells in the TME, including both myeloid and lymphoid lineage cells. PEFs promote the repolarization of macrophages from the M2 phenotype toward the M1 phenotype, suppress myeloid-derived suppressor cell accumulation (MDSCs), and facilitate the differentiation of dendritic cells (DCs) into p-DCs and c-DCs. Furthermore, PEFs enhance the differentiation of T lymphocytes into CD4^+^ and CD8^+^ subsets, and activate B cells and NK cells. Collectively, PEFs drive a shift from an immunosuppressive TME toward an immunostimulatory state. This is characterized by increased pro-inflammatory cytokine production and decreased anti-inflammatory factors, thereby regulating antitumor immunity.

PEFs (IRE and nsPEF) drive anti-tumor responses by systemically reshaping the myeloid immune landscape: They significantly reduce MDSCs proportions (19, 20) and inhibit CCR2^+^TAM-mediated immunosuppression (104), while driving the sphingolipid metabolism-mediated differentiation of Ly6c2^+^ monocytes into dendritic cells (53). Dendritic cells (DCs) act as central hubs—IRE combined with VMT/CaO_2_ NSs captures tumor antigens to form *in situ* vaccines that promote DC migration (105); a substantial intraprocedural resistance drop (large ΔR) upregulates the CD80 costimulatory molecule on cDC1s (106); PD-L1 blockade specifically activates cDC2s to enhance antigen presentation (36); and nsPEF activates the NLRP3 inflammasome to trigger IL-1β release (107)—collectively strengthening myeloid immune initiation. Therefore, in myeloid cells, PEFs primarily modulate MDSCs through the NLRP3/IL-1β signaling pathway and regulate tumor-associated macrophages via the CCLs/CCR2 signaling axis (**Figure 3**).

In the lymphoid compartment, PEFs induce profound responses: Enhanced CD8^+^ T-cell infiltration with dual Ki-67^+^/PD-1^+^ activation (106, 108); sustained central memory T cells (Tcm) strongly correlating with recurrence-free survival (109); CD4^+^ T-cell polarization toward IFN-γ^+^ Th1/Th17 phenotypes (36), expandable via PD-L1/IL-6 dual blockade to amplify the Th1-NK cell axis (36); alongside early NK-cell expansion (109) and increased B-cell infiltration (51, 76) synergistically counteracting Treg-mediated immunosuppression (20, 108). Synergistic strategies further augment efficacy: PD-1/CTLA-4 blockade remodels cytokine profiles and T-cell function (20, 51); oncolytic viruses (OVs) recruit CTLs to intensify local attack (110); and pH-responsive hydrogel microspheres promote cDC1 migration to lymph nodes (111). Thus, it is plausible that PEFs regulate T cells of the lymphoid lineage via the IFN-γ/IFNR and IL-6/PD-L1 signaling axes (**Figure 3**).

In summary, PEFs transform “cold” tumors into immune-permissive states (31, 36, 111) through myeloid reprogramming (enhanced antigen presentation/suppressive cell clearance) and lymphoid activation (effector-memory cell expansion). Their efficacy is modulated by intraprocedural resistance dynamics (ΔR), sphingolipid metabolism, and inflammasome pathways, establishing a novel “local ablation-systemic immunity” paradigm for solid tumors.

## 6 Prospects

PEFs offers significant advantages in tumor therapy, positioning it as a promising tool for both treatment and research (**Table 2**). A key strength is its minimally invasive nature, requiring no major surgery or incisions; instead, electrodes are inserted directly into the tumor area, reducing procedural risks and patient discomfort. The high selectivity of PEFs enables precise targeting of specific cells or tissues through parameter adjustment, sparing surrounding healthy structures (112). Its versatility further enhances utility–PEFs can directly kill or inhibit cancer cells, modulate immune responses, and enhance drug delivery efficacy, supporting applications across diverse therapeutic contexts. Rapid effectiveness is another critical advantage, with tumors often showing swift regression post-treatment, a feature particularly valuable for acute conditions or urgent symptom management. Finally, the adjustability of PEFs parameters allows customization to tumor-specific requirements, facilitating personalized treatment optimization.

**Table 2:** The application of PEFs for cancers in clinical trials (https://clinicaltrials.gov/search?cond=Tumor&intr=IRE&viewType=Table).

NCT number	Tumor type	Interventions	Status	Phase	Study results	Completion date	Outcome measures	Locations
NCT03180437	♦ Pancreatic cancer	Procedure: IRE surgery Biological: IRE plus γδT cells	Completed	Phase 1 Phase 2	Interventional	2019/6/15	♦ PFS ♦ OS Tumor size	China
NCT01442324	♦ Metastatic liver cancer ♦ Cholangiocarcinoma ♦ Neoplasm metastasis	Procedure: IRE	Unknown	Not Applicable	Interventional	–	♦ Effectiveness of IRE for the treatment of metastatic liver cancer or cholangiocarcinoma. ♦ Safety ♦ Time to *in situ* recurrence	Italy
NCT03769753	♦ Cholangiocarcinoma	Device: IRE Device	Completed	Phase 2	Interventional	2021/8/16	♦ Number of patients experiencing a clinically relevant complications	United States
NCT02822716	♦ Hepatic Malignant Tumors ♦ Pancreatic Cancer	Procedure: IRE treatment Device: Ultrasound	Terminated	Not Applicable	Interventional	2020/9/23	♦ Radiological assessment	China
NCT02828865	♦ Hepatocellular Carcinoma ♦ Metastatic Liver Cancers	Device: IRE system	Completed	Not Applicable	Interventional	2020/5/27	♦ Tumor response ♦ Change of Eastern Cooperative Oncology Group (ECOG) evaluation ♦ Change of vital signs	China
NCT02718859	♦ Pancreatic cancer	Biological: NK cells Procedure: IRE	Completed	Phase 1 Phase 2	Interventional	2019/3/1	♦ Relief degree ♦ PFS ♦ OS	China
NCT03069599	♦ Pancreatic cancer	Procedure: IRE	Terminated	–	Observational	2020/2/25	♦ Immunological outcome	Switzerland
NCT02791503	♦ Pancreatic neoplasm	Procedure: IRE Procedure: Stereotactic ablative radiotherapy (SABR)	Completed	Not Applicable	Interventional	2022/9	♦ Overall survival ♦ Progression free survival ♦ uPFS	Netherlands
NCT06205849	♦ Pancreatic cancer	Drug: IRE + intratumoral mitazalimab (CD40 antibody) injection Device: NanoKnife	Recruiting	Phase 1	Interventional	2029/8	♦ Safety and tolerability ♦ PFS ♦ OS	United States
NCT06677762	♦ Pancreatic cancer	Procedure: IRE	Not yet recruiting	–	Observational	2025/6/30	♦ OS ♦ PFS ♦ ORR	China
NCT06047015	♦ Liver metastasis colon cancer	Combination Product: IRE plus checkpoint inhibitor Combination Product: IRE plus checkpoint inhibitor plus CpG-ODN	Not yet recruiting	Phase 1 Phase 2	Interventional	2028/12	♦ Complications ♦ Complications ♦ Abscopal effect: percent change in non-treated colorectal liver metastasis.	Canada
NCT06451445	♦ Prostate cancer	Device: IRE (NanoKnife)	Recruiting	Not Applicable	Interventional	2033/5	♦ Rate of negative in-field biopsy at 12 months ♦ Incidence of adverse events by type and CTCAE v5.0 severity through 12 months ♦ Evaluation of clinically significant prostate cancer rate of development outside the ablation zone	Canada
NCT02335827	♦ Renal tumor	Procedure: IRE	Completed	Not Applicable	Interventional	2021/1/1	♦ adverse effects ♦ QoL ♦ procedural compliance	China
NCT05345444	♦ Prostate cancer	Device: IRE & MRgRT	Active, not recruiting	Not Applicable	Interventional	2027/4	♦ Feasibility as measured by percentage of subjects assessed at 12 weeks post-IRE ♦ Feasibility as measured by percentage of subjects assessed at 6 weeks post-MRgRT within 1 year ♦ Number of treatment-related adverse events	United States
NCT02343835	♦ Prostate cancer	Device: NanoKnife LEDC system	Completed	Not Applicable	Interventional	2021/5/1	♦ Characterization of the intra-tumoral and systemic immune response to IRE in unresectable pancreatic cancers ♦ Comparison immune response between non-ablated and ablated pancreatic cancer and pre-ablated and post ablated serum	China
NCT05513443	♦ Prostate cancer	Procedure: IRE Procedure: Radical prostatectomy Radiation: Radiation therapy	Recruiting	Not Applicable	Interventional	2026/9/1	♦ Primary outcome in PRIS 1: Urinary continence ♦ Primary outcome in PRIS 2: Irritative urinary symptoms ♦ Erectile dysfunction	Sweden
NCT05555342	♦ Lung tumor ♦ Metastatic cancer	Procedure: IRE ablation Radiation: Radiation therapy	Completed	Early Phase 1	Interventional	2024/10/27	♦ Feasibility, measured as the number of patients completing both IRE and the single fraction of radiation ♦ Number of grade 3-5 adverse events ♦ Local failure at the treated site	United States
NCT04972097	♦ Prostate cancer	Device: IRE	Completed	Not Applicable	Interventional	2024/8/14	♦ Rate of negative in-field biopsy at 12 months ♦ Incidence of adverse events by type and CTCAE v5.0 severity through 12 months ♦ Rate of negative in-field biopsy at 12 months as defined by the Delphi consensus criterion	United States
NCT03183232	♦ Lung cancer	Procedure: Cryosurgery or IRE surgery Biological: γδ T cell Other: γδ T cells/A Cryosurgery or IRE	Completed	Phase 1 Phase 2	Interventional	2019/6/15	♦ Relief degree of tumors ♦ PFS ♦ OS	China
NCT03183219	♦ Liver cancer	Procedure: Cryosurgery or IRE surgery Biological: γδ T cell Other: γδ T cells/A Cryosurgery or IRE	Completed	Phase 1 Phase 2	Interventional	2019/6/15	♦ Relief degree of tumors ♦ PFS ♦ OS	China
NCT06378047	♦ Pancreatic cancer ♦ Locally advanced pancreatic cancer	Drug: Pembrolizumab Device: IRE	Recruiting	Phase 1	Interventional	20274/4	♦ Safety and Tolerability ♦ PFS ♦ OS	United States
NCT02340858	♦ Breast cancer	Device: NanoKnife LEDC System	Completed	Not Applicable	Interventional	2021/1/1	♦ Treatment efficacy as measured by modified Response Evaluation Criteria In Solid Tumors (RECIST) criteria by Computed Tomography (CT) or Magnetic Resonance (MR) imaging. ♦ Safety using Common Terminology Criteria for Adverse Events (CTCAE) Version 3.0 criteria.	China
NCT02430636	♦ Stomach Neoplasms	Procedure: IRE Device: NanoKnife	Completed	Not Applicable	Interventional	2021/1/1	♦ Number of participants with Adverse events ♦ Percentage of lesions that show no sign of recurrence 12 months after IRE ♦ A minimum and maximum range of voltage for safe and effective IRE	China

PFS, Progress free survival; OS, Overall Survival; ORR, Objective response rate; uPFS, Untreatable progression-free survival; QoL, Quality of life.

Despite its significant advantages, PEFs face several challenges and limitations in the field of tumor therapy. A primary challenge lies in parameter optimization, where therapeutic efficacy depends on variables such as voltage, frequency, pulse width, and pulse number–identifying the optimal combination for diverse tumor types demands extensive research. Tumor specificity presents another obstacle, as heterogeneous tissue responses to PEFs can compromise treatment consistency and reproducibility, necessitating deeper mechanistic studies (113). Safety concerns also arise, with potential adverse effects including muscle contractions, tissue damage, pain, and inflammation during therapy, underscoring the need for enhanced safety protocols. Limited applicability further constrains PEFs, as certain tumor types exhibit suboptimal responses or unsuitability for this modality, requiring clearer definition of its scope. Finally, the translation to clinical practice remains challenging, demanding large-scale trials to validate efficacy and safety, alongside standardized protocols, equipment, and operational frameworks to ensure reliability (114). Additionally, a more critical analysis of the current limitations in clinical translation is needed, such as the standardization of treatment protocols, the management of off-target effects in complex anatomical locations, and the long-term efficacy and safety data from clinical trials.

In summary, while PEFs hold transformative potential, addressing these practical hurdles through sustained research and technological refinement is essential for advancing their clinical adoption.

## 7 Conclusion

PEFs, as an emerging method for cancer treatment, holds vast potential applications. By disrupting the integrity of tumor cell membranes, PEFs can effectively induce the death of tumor cells and can be combined with other treatment methods to enhance treatment outcomes. Despite some successes in practice, further research is still needed to address the challenges faced by PEFs and to expand its application in clinical practice.
